# Bis[1-benzyl-2-(1,3-thia­zol-4-yl)-1*H*-benzimidazole-κ^2^ *N* ^2^,*N* ^3^]dichloridocobalt(II)

**DOI:** 10.1107/S1600536812048751

**Published:** 2012-12-05

**Authors:** Hicham Gueddar, Rachid Bouhfid, El Mokhtar Essassi, Nabil El Brahmi, Lahcen El Ammari

**Affiliations:** a Laboratoire de Chimie Organique Hétérocyclique, URAC 21, Pôle de Compétences Pharmacochimie, Université Mohammed V-Agdal, BP 1014 Avenue Ibn Batouta, Rabat, Morocco; b Institute of Nanomaterials and Nanotechnology, MAScIR, Avenue de l’Armée Royale, Rabat, Morocco; c Laboratoire de Chimie Organique Hétérocyclique, URAC 21, Faculté des Sciences, Université Mohammed V-Agdal, Avenue Ibn Battouta, BP 1014, Rabat, Morocco; d Laboratoire de Chimie de Coordination, Équipe Dendrimères et Hétérochimie, Toulouse, France; e Laboratoire de Chimie du Solide Appliquée, Faculté des Sciences, Université Mohammed V-Agdal, Avenue Ibn Battouta, BP 1014, Rabat, Morocco

## Abstract

In the title compound, [CoCl_2_(C_17_H_13_N_3_S)_2_], the Co^II^ atom exhibits a distorted octa­hedral coordination geometry involving two chloride ligands, one of which is split over two positions [refined site-occupancy ratio = 0.847 (18):0.153 (18)], and four N-atom donors from two 1-benzyl-2-(1,3-thia­zol-4-yl)-1*H*-benzimidazole ligands. The two chelate rings including the Co^II^ atom are essentially planar, the maximum deviations from the mean planes being 0.080 (2) and 0.046 (2) Å; the dihedral angle between them is 74.1 (1)°. In both ligands, the thia­zole and benzimidazole rings are nearly coplanar, as indicated by the dihedral angles between their planes of 1.16 (8) and 6.29 (7)°. Each pendant benzene ring is almost perpendicular to the benzimidazole mol­ecule to which it is attached; the dihedral angles between their planes are 75.94 (9) and 75.55 (10)°. The crystal structure is stabilized by non-classical C—H⋯Cl hydrogen bonding forming a three-dimensional network.

## Related literature

For background of the biochemical properties of thia­bendazole [2-(4′-thia­zol­yl)benzimidazole], see: Devereux *et al.* (2007 ▶); Kowala *et al.* (1971 ▶); Yan-Jua & Guang-Ganga (2009 ▶).
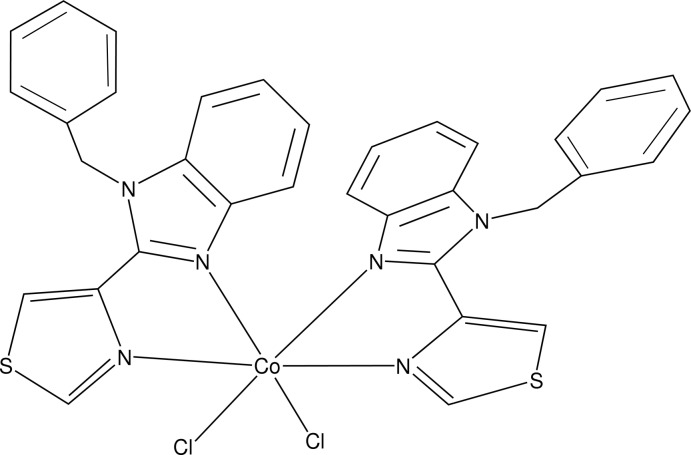


## Experimental

### 

#### Crystal data

[CoCl_2_(C_17_H_13_N_3_S)_2_]
*M* *_r_* = 712.56
Triclinic, 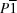

*a* = 10.1311 (3) Å
*b* = 11.9582 (4) Å
*c* = 14.2633 (5) Å
α = 76.033 (3)°
β = 75.536 (3)°
γ = 69.707 (3)°
*V* = 1546.43 (9) Å^3^
*Z* = 2
Mo *K*α radiation
μ = 0.90 mm^−1^
*T* = 193 K
0.40 × 0.40 × 0.20 mm

#### Data collection

Bruker Kappa APEXII Quazar area-detector diffractometer
Absorption correction: multi-scan (*SADABS*; Bruker, 2009 ▶) *T*_min_ = 0.682, *T*_max_ = 0.840
11578 measured reflections
6284 independent reflections
5601 reflections with *I* > 2σ(*I*)
*R*_int_ = 0.011

#### Refinement

*R*[*F* ^2^ > 2σ(*F* ^2^)] = 0.030
*wR*(*F* ^2^) = 0.080
*S* = 1.05
6284 reflections
417 parameters
H-atom parameters constrained
Δρ_max_ = 0.95 e Å^−3^
Δρ_min_ = −0.68 e Å^−3^

### 

Data collection: *APEX2* (Bruker, 2009 ▶); cell refinement: *SAINT* (Bruker, 2009 ▶); data reduction: *SAINT*; program(s) used to solve structure: *SHELXS97* (Sheldrick, 2008 ▶); program(s) used to refine structure: *SHELXL97* (Sheldrick, 2008 ▶); molecular graphics: *ORTEP-3 for Windows* (Farrugia, 2012 ▶); software used to prepare material for publication: *PLATON* (Spek, 2009 ▶) and *publCIF* (Westrip, 2010 ▶).

## Supplementary Material

Crystal structure: contains datablock(s) I, global. DOI: 10.1107/S1600536812048751/bt6860sup1.cif

Structure factors: contains datablock(s) I. DOI: 10.1107/S1600536812048751/bt6860Isup2.hkl

Additional supplementary materials:  crystallographic information; 3D view; checkCIF report

## Figures and Tables

**Table 1:** Hydrogen-bond geometry (Å, °)

*D*—H⋯*A*	*D*—H	H⋯*A*	*D*⋯*A*	*D*—H⋯*A*
C11—H11*A*⋯Cl2*A* ^i^	0.99	2.77	3.693 (2)	155
C14—H14⋯Cl1^ii^	0.95	2.69	3.584 (2)	157

Symmetry codes: (i) 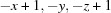; (ii) 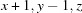.

**Fig. 1.**
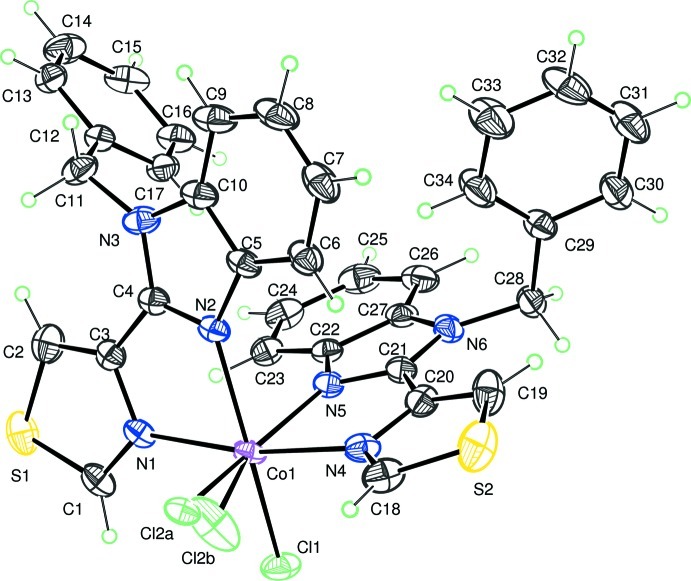
Molecular structure of the title compound with the atom-labelling scheme. Displacement ellipsoids are drawn at the 50% probability level. H atoms are represented as small circles.

## supplementary crystallographic information

### Comment

The thiabendazole or (2-(4'-thiazolyl)benzimidazole), is an antimicrobial drug belonging to the benzimidazole derivatives which are ubiquitous in biology and biomedicine (Devereux *et al.* 2007), Beside its biological properties, thiabendazole is an effective ligand to coordinate transition metal ions (Kowala *et al.*, 1971; Yan-Jua and Guang-Ganga, 2009).

The crystal structure of the title compound, show that the Co^II^ ion adopts a distorted octahedral coordination arising from two bidentate ligands and a two Cl^-^ anion of which one (Cl2) is splited over two positions (Cl2a and Cl2b) and four nitrogen donors from the ligands. Indeed, the refined occupancy rate of Cl2a and Cl2b sites shows that the first is occupied at 95 (2) % and the remainder in the second site respectively (Fig.1). The two heterocyclic ligands (S1N1C1C2C3) and (N2N3C4 to C10); (S2N4C18C1920) and (N5N6C21 TO C27) are nearly coplanar with dihedral angles between them of 1.16 (8) ° and 6.26 (9)° respectively. The dihedral angle between the both thiabendazole molecules surrounding the cobalt atom is of 74.1 (1)°. Each benzene ring (C12 to C17 and C29 to C34) is virtually perpendicular to the benzimidazole molecule (N2N3C5 to C10 and N5N6C21 to C27) to which it is fixed and the dihedral angle between them is 75.94 (9) for the first system and 75.55 (10) for the second.

The crystal strucrure is further stabilized by an intermolecular C—H···Cl no classic hydrogen bonds (Table 2).

### Experimental

Thiabendazole (1.22 g, 6.02 mmol) was dissolved in 20 ml of ethanol, and CoCl_2_.6H_2_O (0.48 g, 3.02 mmol) dissolved in 1 ml of water were added. After 3 days of stirring at room temperature, a single-crystal precipitated and was separated by filtration and dried at 333 K for 24 h.

### Refinement

The highest peak (2.04) and the deepest hole (-1.02) in the final Fourier map are at 0.92 Å and 0.65 Å from Cl2. The refinement of the structure in the space group P1 with a twinning model has not led to the desired result. The splitting of one chlorine position (Cl2) in the centrosymmetric group and the refinement of the occupancy rate of each position has led to a slight improvement of the refinement and there is no longer remains electronic density near the chlorine. Indeed, the refined occupancy rate of Cl2a and Cl2b sites shows that the first is occupied at 91.8 (2) % and the remainder in the second site respectively. Now, the highest peak (O.95) and the deepest hole (-0.68) in the final Fourier map are at 0.82 Å and 0.68 Å from Co1. H atoms were located in a difference map and treated as riding with N—H = 0.86 Å, C—H = 0.93 Å (aromatic), and C—H = 0.97 Å (methylene). with *U*_iso_(H) = 1.2 *U*_eq_ (aromatic, methylene).

### Crystal data

**Table:**

[CoCl_2_(C_17_H_13_N_3_S)_2_]	*Z* = 2
*M**_r_* = 712.56	*F*(000) = 730
Triclinic, *P*1	*D*_x_ = 1.530 Mg m^−^^3^
Hall symbol: -p 1	Mo *K*α radiation, λ = 0.71073 Å
*a* = 10.1311 (3) Å	Cell parameters from 6245 reflections
*b* = 11.9582 (4) Å	θ = 5.1–26.4°
*c* = 14.2633 (5) Å	µ = 0.90 mm^−^^1^
α = 76.033 (3)°	*T* = 193 K
β = 75.536 (3)°	Block, pink
γ = 69.707 (3)°	0.40 × 0.40 × 0.20 mm
*V* = 1546.43 (9) Å^3^	

### Data collection

**Table:**

Bruker Kappa APEXII Quazar area-detector diffractometer	6284 independent reflections
Radiation source: microfocus sealed tube	5601 reflections with *I* > 2σ(*I*)
Multilayer optics monochromator	*R*_int_ = 0.011
φ and ω scans	θ_max_ = 26.4°, θ_min_ = 5.1°
Absorption correction: multi-scan (*SADABS*; Bruker, 2009)	*h* = −12→11
*T*_min_ = 0.682, *T*_max_ = 0.840	*k* = −14→14
11578 measured reflections	*l* = −17→16

### Refinement

**Table:**

Refinement on *F*^2^	Secondary atom site location: difference Fourier map
Least-squares matrix: full	Hydrogen site location: inferred from neighbouring sites
*R*[*F*^2^ > 2σ(*F*^2^)] = 0.030	H-atom parameters constrained
*wR*(*F*^2^) = 0.080	*w* = 1/[σ^2^(*F*_o_^2^) + (0.0362*P*)^2^ + 1.3427*P*] where *P* = (*F*_o_^2^ + 2*F*_c_^2^)/3
*S* = 1.05	(Δ/σ)_max_ = 0.001
6284 reflections	Δρ_max_ = 0.95 e Å^−^^3^
417 parameters	Δρ_min_ = −0.68 e Å^−^^3^
0 restraints	Extinction correction: *SHELXL97* (Sheldrick, 2008), Fc^*^=kFc[1+0.001xFc^2^λ^3^/sin(2θ)]^-1/4^
Primary atom site location: structure-invariant direct methods	Extinction coefficient: 0.0040 (5)

### Special details

**Table:**

Geometry. All e.s.d.'s (except the e.s.d. in the dihedral angle between two l.s. planes) are estimated using the full covariance matrix. The cell e.s.d.'s are taken into account individually in the estimation of e.s.d.'s in distances, angles and torsion angles; correlations between e.s.d.'s in cell parameters are only used when they are defined by crystal symmetry. An approximate (isotropic) treatment of cell e.s.d.'s is used for estimating e.s.d.'s involving l.s. planes.
Refinement. Refinement of *F*^2^ against all reflections. The weighted *R*-factor *wR* and goodness of fit *S* are based on *F*^2^, conventional *R*-factors *R* are based on *F*, with *F* set to zero for negative *F*^2^. The threshold expression of *F*^2^ > 2σ(*F*^2^) is used only for calculating *R*-factors(gt) *etc*. and is not relevant to the choice of reflections for refinement. *R*-factors based on *F*^2^ are statistically about twice as large as those based on *F*, and *R*- factors based on all data will be even larger.

### Fractional atomic coordinates and isotropic or equivalent isotropic displacement parameters (Å^2^)

**Table:**

	*x*	*y*	*z*	*U*_iso_*/*U*_eq_	Occ. (<1)
C1	0.6603 (2)	0.37010 (19)	0.43804 (14)	0.0281 (4)	
H1	0.6306	0.4540	0.4409	0.034*	
C2	0.7805 (2)	0.1569 (2)	0.44040 (15)	0.0327 (4)	
H2	0.8419	0.0759	0.4434	0.039*	
C3	0.64845 (19)	0.19650 (17)	0.41692 (13)	0.0223 (4)	
C4	0.56253 (19)	0.13517 (16)	0.39322 (13)	0.0214 (4)	
C5	0.3835 (2)	0.11633 (17)	0.35002 (13)	0.0236 (4)	
C6	0.2527 (2)	0.13165 (19)	0.32592 (14)	0.0289 (4)	
H6	0.1811	0.2082	0.3214	0.035*	
C7	0.2314 (3)	0.0305 (2)	0.30877 (15)	0.0366 (5)	
H7	0.1435	0.0381	0.2918	0.044*	
C8	0.3360 (3)	−0.0824 (2)	0.31586 (16)	0.0388 (5)	
H8	0.3176	−0.1493	0.3027	0.047*	
C9	0.4642 (3)	−0.09957 (19)	0.34126 (15)	0.0346 (5)	
H9	0.5349	−0.1765	0.3467	0.041*	
C10	0.4850 (2)	0.00220 (17)	0.35870 (13)	0.0260 (4)	
C11	0.7263 (2)	−0.08140 (17)	0.40841 (15)	0.0301 (4)	
H11A	0.7035	−0.1588	0.4266	0.036*	
H11B	0.7546	−0.0691	0.4659	0.036*	
C12	0.8512 (2)	−0.09147 (16)	0.32447 (13)	0.0226 (4)	
C13	0.9687 (2)	−0.19429 (17)	0.33241 (15)	0.0292 (4)	
H13	0.9665	−0.2561	0.3884	0.035*	
C14	1.0883 (2)	−0.20696 (19)	0.25942 (18)	0.0364 (5)	
H14	1.1685	−0.2774	0.2651	0.044*	
C15	1.0916 (2)	−0.11754 (19)	0.17831 (17)	0.0345 (5)	
H15	1.1748	−0.1259	0.1285	0.041*	
C16	0.9753 (2)	−0.01644 (18)	0.16905 (15)	0.0299 (4)	
H16	0.9776	0.0445	0.1124	0.036*	
C17	0.8548 (2)	−0.00304 (17)	0.24201 (14)	0.0256 (4)	
H17	0.7744	0.0671	0.2355	0.031*	
C18	0.0728 (2)	0.47264 (19)	0.31938 (17)	0.0328 (4)	
H18	0.0335	0.4909	0.3837	0.039*	
C19	0.1212 (2)	0.4361 (2)	0.15296 (17)	0.0370 (5)	
H19	0.1224	0.4249	0.0890	0.044*	
C20	0.2387 (2)	0.41930 (17)	0.18986 (15)	0.0258 (4)	
C21	0.3907 (2)	0.38422 (16)	0.14785 (13)	0.0221 (4)	
C22	0.61612 (19)	0.34029 (15)	0.14499 (13)	0.0211 (4)	
C23	0.7512 (2)	0.31937 (16)	0.16493 (14)	0.0251 (4)	
H23	0.7639	0.3202	0.2285	0.030*	
C24	0.8657 (2)	0.29749 (18)	0.08846 (16)	0.0314 (4)	
H24	0.9593	0.2821	0.0999	0.038*	
C25	0.8473 (2)	0.29740 (19)	−0.00575 (16)	0.0349 (5)	
H25	0.9291	0.2818	−0.0565	0.042*	
C26	0.7144 (2)	0.31922 (18)	−0.02687 (15)	0.0308 (4)	
H26	0.7020	0.3200	−0.0909	0.037*	
C27	0.5995 (2)	0.34003 (16)	0.05055 (14)	0.0236 (4)	
C28	0.3882 (2)	0.37895 (18)	−0.02866 (14)	0.0287 (4)	
H28A	0.4548	0.3952	−0.0904	0.034*	
H28B	0.3003	0.4492	−0.0273	0.034*	
C29	0.3500 (2)	0.26816 (18)	−0.02967 (14)	0.0268 (4)	
C30	0.3025 (3)	0.2664 (2)	−0.11220 (17)	0.0370 (5)	
H30	0.2987	0.3317	−0.1658	0.044*	
C31	0.2607 (3)	0.1699 (2)	−0.11673 (18)	0.0454 (6)	
H31	0.2260	0.1699	−0.1728	0.055*	
C32	0.2692 (3)	0.0738 (2)	−0.04062 (18)	0.0462 (6)	
H32	0.2412	0.0071	−0.0442	0.055*	
C33	0.3181 (3)	0.0742 (2)	0.04040 (19)	0.0531 (7)	
H33	0.3247	0.0075	0.0929	0.064*	
C34	0.3580 (3)	0.1720 (2)	0.04607 (17)	0.0441 (6)	
H34	0.3912	0.1721	0.1027	0.053*	
N1	0.58202 (16)	0.31923 (14)	0.41412 (11)	0.0224 (3)	
N2	0.43504 (16)	0.19849 (13)	0.37231 (11)	0.0208 (3)	
N3	0.59824 (17)	0.01613 (14)	0.38752 (12)	0.0253 (3)	
N4	0.20969 (16)	0.43936 (14)	0.28531 (12)	0.0253 (3)	
N5	0.48317 (16)	0.36795 (13)	0.20426 (11)	0.0208 (3)	
N6	0.45439 (17)	0.36890 (14)	0.05423 (11)	0.0240 (3)	
S1	0.82050 (6)	0.27380 (6)	0.46385 (4)	0.03746 (14)	
S2	−0.02965 (6)	0.48051 (6)	0.23841 (5)	0.04537 (16)	
Cl1	0.42867 (5)	0.58768 (4)	0.30588 (4)	0.03220 (12)	
Cl2A	0.23855 (17)	0.40676 (12)	0.5152 (2)	0.0232 (5)	0.847 (18)
Cl2B	0.215 (2)	0.444 (4)	0.485 (2)	0.060 (6)	0.153 (18)
Co1	0.39016 (2)	0.39444 (2)	0.355093 (18)	0.02006 (8)	

### Atomic displacement parameters (Å^2^)

**Table:**

	*U*^11^	*U*^22^	*U*^33^	*U*^12^	*U*^13^	*U*^23^
C1	0.0262 (9)	0.0369 (11)	0.0278 (10)	−0.0143 (8)	−0.0031 (8)	−0.0125 (8)
C2	0.0277 (10)	0.0361 (11)	0.0330 (11)	−0.0096 (8)	−0.0084 (8)	−0.0009 (9)
C3	0.0231 (9)	0.0251 (9)	0.0182 (8)	−0.0092 (7)	−0.0003 (7)	−0.0038 (7)
C4	0.0232 (9)	0.0223 (9)	0.0179 (8)	−0.0088 (7)	0.0017 (7)	−0.0049 (7)
C5	0.0313 (10)	0.0254 (9)	0.0181 (8)	−0.0162 (8)	0.0022 (7)	−0.0066 (7)
C6	0.0363 (11)	0.0312 (10)	0.0258 (9)	−0.0186 (9)	−0.0055 (8)	−0.0046 (8)
C7	0.0499 (13)	0.0449 (13)	0.0290 (10)	−0.0316 (11)	−0.0084 (9)	−0.0051 (9)
C8	0.0610 (15)	0.0334 (11)	0.0332 (11)	−0.0302 (11)	−0.0008 (10)	−0.0107 (9)
C9	0.0485 (13)	0.0253 (10)	0.0311 (10)	−0.0185 (9)	0.0061 (9)	−0.0100 (8)
C10	0.0327 (10)	0.0243 (9)	0.0213 (9)	−0.0140 (8)	0.0049 (7)	−0.0070 (7)
C11	0.0350 (11)	0.0207 (9)	0.0263 (10)	−0.0045 (8)	−0.0004 (8)	0.0001 (7)
C12	0.0255 (9)	0.0199 (9)	0.0240 (9)	−0.0066 (7)	−0.0052 (7)	−0.0066 (7)
C13	0.0319 (10)	0.0217 (9)	0.0356 (11)	−0.0061 (8)	−0.0129 (8)	−0.0041 (8)
C14	0.0229 (10)	0.0282 (10)	0.0582 (14)	−0.0025 (8)	−0.0070 (9)	−0.0161 (10)
C15	0.0291 (10)	0.0311 (11)	0.0464 (12)	−0.0140 (9)	0.0067 (9)	−0.0197 (9)
C16	0.0362 (11)	0.0258 (10)	0.0290 (10)	−0.0139 (8)	0.0027 (8)	−0.0092 (8)
C17	0.0286 (10)	0.0204 (9)	0.0255 (9)	−0.0053 (7)	−0.0027 (8)	−0.0051 (7)
C18	0.0215 (9)	0.0289 (10)	0.0474 (12)	−0.0078 (8)	−0.0027 (9)	−0.0087 (9)
C19	0.0327 (11)	0.0465 (13)	0.0368 (11)	−0.0206 (10)	−0.0141 (9)	0.0044 (10)
C20	0.0253 (9)	0.0224 (9)	0.0333 (10)	−0.0118 (7)	−0.0085 (8)	−0.0014 (8)
C21	0.0259 (9)	0.0180 (8)	0.0262 (9)	−0.0109 (7)	−0.0057 (7)	−0.0034 (7)
C22	0.0242 (9)	0.0151 (8)	0.0238 (9)	−0.0072 (7)	−0.0014 (7)	−0.0040 (7)
C23	0.0246 (9)	0.0215 (9)	0.0281 (9)	−0.0078 (7)	−0.0034 (7)	−0.0029 (7)
C24	0.0247 (10)	0.0253 (10)	0.0404 (11)	−0.0075 (8)	−0.0003 (8)	−0.0047 (8)
C25	0.0350 (11)	0.0267 (10)	0.0353 (11)	−0.0078 (8)	0.0089 (9)	−0.0097 (8)
C26	0.0409 (11)	0.0238 (9)	0.0269 (10)	−0.0107 (8)	0.0016 (8)	−0.0093 (8)
C27	0.0319 (10)	0.0150 (8)	0.0249 (9)	−0.0093 (7)	−0.0033 (7)	−0.0037 (7)
C28	0.0415 (11)	0.0254 (10)	0.0246 (9)	−0.0142 (8)	−0.0125 (8)	−0.0015 (7)
C29	0.0308 (10)	0.0275 (10)	0.0268 (9)	−0.0125 (8)	−0.0048 (8)	−0.0080 (8)
C30	0.0461 (13)	0.0391 (12)	0.0344 (11)	−0.0170 (10)	−0.0156 (10)	−0.0072 (9)
C31	0.0549 (15)	0.0564 (15)	0.0425 (13)	−0.0287 (12)	−0.0153 (11)	−0.0164 (11)
C32	0.0615 (16)	0.0519 (15)	0.0437 (13)	−0.0398 (13)	0.0016 (11)	−0.0201 (11)
C33	0.094 (2)	0.0456 (14)	0.0383 (13)	−0.0476 (15)	−0.0124 (13)	−0.0008 (11)
C34	0.0774 (18)	0.0400 (13)	0.0310 (11)	−0.0352 (13)	−0.0170 (11)	−0.0023 (9)
N1	0.0217 (7)	0.0267 (8)	0.0225 (7)	−0.0105 (6)	−0.0014 (6)	−0.0094 (6)
N2	0.0241 (8)	0.0209 (7)	0.0199 (7)	−0.0105 (6)	−0.0005 (6)	−0.0064 (6)
N3	0.0273 (8)	0.0198 (8)	0.0264 (8)	−0.0089 (6)	0.0030 (6)	−0.0050 (6)
N4	0.0198 (7)	0.0219 (8)	0.0358 (9)	−0.0076 (6)	−0.0030 (6)	−0.0085 (7)
N5	0.0223 (7)	0.0189 (7)	0.0233 (7)	−0.0089 (6)	−0.0036 (6)	−0.0042 (6)
N6	0.0313 (8)	0.0212 (7)	0.0246 (8)	−0.0126 (6)	−0.0072 (6)	−0.0042 (6)
S1	0.0308 (3)	0.0532 (3)	0.0371 (3)	−0.0197 (2)	−0.0136 (2)	−0.0056 (2)
S2	0.0221 (3)	0.0555 (4)	0.0574 (4)	−0.0146 (2)	−0.0140 (2)	0.0036 (3)
Cl1	0.0315 (2)	0.0200 (2)	0.0489 (3)	−0.00923 (18)	−0.0090 (2)	−0.0089 (2)
Cl2A	0.0228 (4)	0.0248 (8)	0.0240 (7)	−0.0103 (4)	0.0029 (3)	−0.0110 (4)
Cl2B	0.039 (4)	0.108 (13)	0.050 (8)	−0.038 (7)	0.011 (5)	−0.041 (10)
Co1	0.01664 (13)	0.02009 (13)	0.02660 (14)	−0.00703 (9)	−0.00087 (9)	−0.01077 (10)

### Geometric parameters (Å, º)

**Table:**

C1—N1	1.298 (2)	C19—S2	1.710 (2)
C1—S1	1.701 (2)	C19—H19	0.9500
C1—H1	0.9500	C20—N4	1.381 (3)
C2—C3	1.354 (3)	C20—C21	1.456 (3)
C2—S1	1.708 (2)	C21—N5	1.318 (2)
C2—H2	0.9500	C21—N6	1.356 (2)
C3—N1	1.382 (2)	C22—N5	1.377 (2)
C3—C4	1.456 (3)	C22—C23	1.392 (3)
C4—N2	1.317 (2)	C22—C27	1.400 (3)
C4—N3	1.359 (2)	C23—C24	1.378 (3)
C5—N2	1.389 (2)	C23—H23	0.9500
C5—C6	1.392 (3)	C24—C25	1.402 (3)
C5—C10	1.394 (3)	C24—H24	0.9500
C6—C7	1.385 (3)	C25—C26	1.376 (3)
C6—H6	0.9500	C25—H25	0.9500
C7—C8	1.397 (3)	C26—C27	1.388 (3)
C7—H7	0.9500	C26—H26	0.9500
C8—C9	1.369 (3)	C27—N6	1.379 (2)
C8—H8	0.9500	C28—N6	1.460 (2)
C9—C10	1.392 (3)	C28—C29	1.508 (3)
C9—H9	0.9500	C28—H28A	0.9900
C10—N3	1.383 (3)	C28—H28B	0.9900
C11—N3	1.452 (2)	C29—C34	1.370 (3)
C11—C12	1.502 (3)	C29—C30	1.386 (3)
C11—H11A	0.9900	C30—C31	1.380 (3)
C11—H11B	0.9900	C30—H30	0.9500
C12—C17	1.381 (3)	C31—C32	1.372 (4)
C12—C13	1.388 (3)	C31—H31	0.9500
C13—C14	1.376 (3)	C32—C33	1.368 (3)
C13—H13	0.9500	C32—H32	0.9500
C14—C15	1.375 (3)	C33—C34	1.388 (3)
C14—H14	0.9500	C33—H33	0.9500
C15—C16	1.371 (3)	C34—H34	0.9500
C15—H15	0.9500	N1—Co1	2.1297 (15)
C16—C17	1.382 (3)	N2—Co1	2.1901 (15)
C16—H16	0.9500	N4—Co1	2.1429 (16)
C17—H17	0.9500	N5—Co1	2.1739 (15)
C18—N4	1.300 (3)	Cl1—Co1	2.3855 (5)
C18—S2	1.703 (2)	Cl2A—Cl2B	0.57 (5)
C18—H18	0.9500	Cl2A—Co1	2.4181 (19)
C19—C20	1.352 (3)	Cl2B—Co1	2.261 (9)
			
N1—C1—S1	114.29 (16)	C26—C25—H25	119.0
N1—C1—H1	122.9	C24—C25—H25	119.0
S1—C1—H1	122.9	C25—C26—C27	116.32 (19)
C3—C2—S1	110.12 (16)	C25—C26—H26	121.8
C3—C2—H2	124.9	C27—C26—H26	121.8
S1—C2—H2	124.9	N6—C27—C26	131.34 (18)
C2—C3—N1	114.08 (17)	N6—C27—C22	106.17 (16)
C2—C3—C4	132.60 (18)	C26—C27—C22	122.42 (18)
N1—C3—C4	113.32 (16)	N6—C28—C29	114.23 (16)
N2—C4—N3	113.06 (16)	N6—C28—H28A	108.7
N2—C4—C3	119.16 (16)	C29—C28—H28A	108.7
N3—C4—C3	127.76 (17)	N6—C28—H28B	108.7
N2—C5—C6	130.57 (18)	C29—C28—H28B	108.7
N2—C5—C10	109.28 (17)	H28A—C28—H28B	107.6
C6—C5—C10	120.10 (17)	C34—C29—C30	119.26 (19)
C7—C6—C5	117.1 (2)	C34—C29—C28	123.75 (18)
C7—C6—H6	121.4	C30—C29—C28	116.99 (18)
C5—C6—H6	121.4	C31—C30—C29	120.2 (2)
C6—C7—C8	121.7 (2)	C31—C30—H30	119.9
C6—C7—H7	119.1	C29—C30—H30	119.9
C8—C7—H7	119.1	C32—C31—C30	120.3 (2)
C9—C8—C7	121.93 (19)	C32—C31—H31	119.9
C9—C8—H8	119.0	C30—C31—H31	119.9
C7—C8—H8	119.0	C33—C32—C31	119.8 (2)
C8—C9—C10	116.2 (2)	C33—C32—H32	120.1
C8—C9—H9	121.9	C31—C32—H32	120.1
C10—C9—H9	121.9	C32—C33—C34	120.2 (2)
N3—C10—C9	131.1 (2)	C32—C33—H33	119.9
N3—C10—C5	105.95 (16)	C34—C33—H33	119.9
C9—C10—C5	122.9 (2)	C29—C34—C33	120.3 (2)
N3—C11—C12	114.20 (15)	C29—C34—H34	119.9
N3—C11—H11A	108.7	C33—C34—H34	119.9
C12—C11—H11A	108.7	C1—N1—C3	111.49 (16)
N3—C11—H11B	108.7	C1—N1—Co1	131.34 (14)
C12—C11—H11B	108.7	C3—N1—Co1	116.57 (12)
H11A—C11—H11B	107.6	C4—N2—C5	105.28 (15)
C17—C12—C13	119.35 (18)	C4—N2—Co1	113.82 (12)
C17—C12—C11	123.18 (17)	C5—N2—Co1	139.41 (13)
C13—C12—C11	117.45 (17)	C4—N3—C10	106.42 (16)
C14—C13—C12	120.21 (19)	C4—N3—C11	128.90 (17)
C14—C13—H13	119.9	C10—N3—C11	124.67 (16)
C12—C13—H13	119.9	C18—N4—C20	111.57 (17)
C15—C14—C13	119.97 (19)	C18—N4—Co1	131.45 (15)
C15—C14—H14	120.0	C20—N4—Co1	116.63 (12)
C13—C14—H14	120.0	C21—N5—C22	105.84 (15)
C16—C15—C14	120.30 (19)	C21—N5—Co1	115.38 (12)
C16—C15—H15	119.9	C22—N5—Co1	138.75 (12)
C14—C15—H15	119.9	C21—N6—C27	106.33 (15)
C15—C16—C17	120.08 (19)	C21—N6—C28	128.80 (17)
C15—C16—H16	120.0	C27—N6—C28	124.88 (16)
C17—C16—H16	120.0	C1—S1—C2	89.98 (10)
C12—C17—C16	120.09 (18)	C18—S2—C19	90.14 (10)
C12—C17—H17	120.0	Cl2B—Cl2A—Co1	67.3 (11)
C16—C17—H17	120.0	Cl2A—Cl2B—Co1	99 (2)
N4—C18—S2	114.02 (17)	N1—Co1—N4	169.37 (6)
N4—C18—H18	123.0	N1—Co1—N5	98.02 (6)
S2—C18—H18	123.0	N4—Co1—N5	75.45 (6)
C20—C19—S2	109.95 (17)	N1—Co1—N2	75.58 (6)
C20—C19—H19	125.0	N4—Co1—N2	94.82 (6)
S2—C19—H19	125.0	N5—Co1—N2	79.86 (5)
C19—C20—N4	114.31 (18)	N1—Co1—Cl2B	104.7 (9)
C19—C20—C21	132.2 (2)	N4—Co1—Cl2B	81.7 (8)
N4—C20—C21	113.53 (16)	N5—Co1—Cl2B	157.1 (8)
N5—C21—N6	112.85 (16)	N2—Co1—Cl2B	102.9 (11)
N5—C21—C20	118.58 (17)	N1—Co1—Cl1	91.26 (4)
N6—C21—C20	128.53 (17)	N4—Co1—Cl1	96.61 (4)
N5—C22—C23	130.57 (17)	N5—Co1—Cl1	86.27 (4)
N5—C22—C27	108.81 (16)	N2—Co1—Cl1	159.21 (4)
C23—C22—C27	120.57 (17)	Cl2B—Co1—Cl1	95.9 (9)
C24—C23—C22	117.13 (18)	N1—Co1—Cl2A	93.31 (8)
C24—C23—H23	121.4	N4—Co1—Cl2A	91.82 (8)
C22—C23—H23	121.4	N5—Co1—Cl2A	164.99 (7)
C23—C24—C25	121.7 (2)	N2—Co1—Cl2A	93.56 (5)
C23—C24—H24	119.2	Cl2B—Co1—Cl2A	13.4 (12)
C25—C24—H24	119.2	Cl1—Co1—Cl2A	103.35 (3)
C26—C25—C24	121.90 (19)		

### Hydrogen-bond geometry (Å, º)

**Table:**

*D*—H···*A*	*D*—H	H···*A*	*D*···*A*	*D*—H···*A*
C11—H11*A*···Cl2*A*^i^	0.99	2.77	3.693 (2)	155
C14—H14···Cl1^ii^	0.95	2.69	3.584 (2)	157

Symmetry codes: (i) −*x*+1, −*y*, −*z*+1; (ii) *x*+1, *y*−1, *z*.
